# Membrane-bound chemoreception of bitter bile acids and peptides is mediated by the same subset of bitter taste receptors

**DOI:** 10.1007/s00018-024-05202-6

**Published:** 2024-05-15

**Authors:** Silvia Schaefer, Florian Ziegler, Tatjana Lang, Alexandra Steuer, Antonella Di Pizio, Maik Behrens

**Affiliations:** 1 TUM Graduate School, TUM School of Life Sciences Weihenstephan, Technical University of Munich, Alte Akademie 8, 85354 Freising, Germany; 2 Leibniz Institute for Food Systems Biology at the Technical University of Munich, Lise-Meitner-Strasse 34, 85354 Freising, Germany; 3 Chemoinformatics and Protein Modelling, Technical University of Munich, Freising, Germany

**Keywords:** Bitter taste receptor, Bile acid, Amino acid, Calcium-mobilization assay, Evolution, Molecular modeling

## Abstract

The vertebrate sense of taste allows rapid assessment of the nutritional quality and potential presence of harmful substances prior to ingestion. Among the five basic taste qualities, salty, sour, sweet, umami, and bitter, bitterness is associated with the presence of putative toxic substances and elicits rejection behaviors in a wide range of animals including humans. However, not all bitter substances are harmful, some are thought to be health-beneficial and nutritious. Among those compound classes that elicit a bitter taste although being non-toxic and partly even essential for humans are bitter peptides and l-amino acids. Using functional heterologous expression assays, we observed that the 5 dominant human bitter taste receptors responsive to bitter peptides and amino acids are activated by bile acids, which are notorious for their extreme bitterness. We further demonstrate that the cross-reactivity of bitter taste receptors for these two different compound classes is evolutionary conserved and can be traced back to the amphibian lineage. Moreover, we show that the cross-detection by some receptors relies on “structural mimicry” between the very bitter peptide l-Trp-Trp-Trp and bile acids, whereas other receptors exhibit a phylogenetic conservation of this trait. As some bile acid-sensitive bitter taste receptor genes fulfill dual-roles in gustatory and non-gustatory systems, we suggest that the phylogenetic conservation of the rather surprising cross-detection of the two substance classes could rely on a gene-sharing-like mechanism in which the non-gustatory function accounts for the bitter taste response to amino acids and peptides.

**Supplementary Information:**

The online version contains supplementary material available at 10.1007/s00018-024-05202-6.

## Introduction

The sense of taste guides food consumption towards nutritionally relevant food sources and away from potentially hazardous substances [1]. Of the five basic taste qualities detected in humans sweet and umami indicate the presence of energy, salty serves a role in electrolyte homeostasis, whereas sour and, especially bitter, hint at the presence of potentially harmful substances [1]. In general, a strong bitterness is coupled to aversive behavior to avoid the ingestion of putative toxic compounds, although a strict relationship between bitterness and toxicity is lacking [2, 3]. The perception of the five basic taste qualities salty, sour, sweet, umami, and bitter occurs in the oral cavity, where specialized sensory cells grouped together in taste buds facilitate detection of taste stimuli [4]. The detection of bitter stimuli relies on receptors of the taste 2 receptor (TAS2R in human, Tas2r in mouse, T2R frequently used alternative gene symbol) family of G protein-coupled receptors [5–8]. The number of functional bitter taste receptor genes fluctuates considerably among different species ranging from e.g. zero to few in dolphins and chicken [9, 10] to several dozens in some amphibian [9, 11], reptilian [12] and bony fish species [13–15]. The large deviations in the bitter taste receptor repertoires of different animals attests to the extraordinary dynamic evolution and the variability in nutritional habits [10, 16, 17]. Not only the number of bitter taste receptors varies considerably, but also the recognition spectra of individual receptors. In human, TAS2Rs can be classified into broadly, intermediately, and narrowly tuned receptors [18]. Moreover, two of the ~ 25 functional TAS2Rs possess pronounced selectivity for chemically similar compound classes [19, 20]. Previous research suggested that in some instances bitter taste receptor repertoire sizes and tuning breadths of individual receptors may represent two sides of a coin, as e.g. the small bitter taste receptor repertoire in chicken consists of only broadly tuned receptors and thus, is still able to support the recognition of a large number of chemically diverse bitter substances [9].

Taste receptor expression is not limited to the gustatory system and therefore additional physiological roles have been proposed for sweet, umami, and bitter taste receptors (for recent reviews see [21–23]). Bitter taste receptors in the respiratory system were shown to be expressed in solitary chemosensory cells in the upper airways where they are involved in pathogen defense [24], in ciliated cells their activation results in elevated beat frequency [25] as well as in smooth muscle cells that respond with relaxation upon bitter stimulation [26]. Also within the gastrointestinal tract, bitter taste receptors were reported to contribute to defense reactions and metabolic regulation [21–23]. Whereas one can imagine that the bitter taste receptors in the mentioned tissues are targeted by the same xenobiotics triggering responses in the oral cavity, other tissues with proven bitter taste receptor expression such as heart [27, 28] and brain [29] are inaccessible from the outside world and hence, endogenously synthesized agonists for those receptors may exist [16].

Previous functional characterizations of human bitter taste receptors revealed a group of at least five TAS2Rs being responsive to some l-amino acids and peptides [30]. More recently, we found that the same subset of human TAS2Rs is also sensitive to a variety of bile acids [31]. This rather surprising finding motivated us to investigate this astonishing coincidence in more detail by comprehensive functional analyses and molecular modeling to elucidate the structural basis for this phenomenon and to study the evolutionary conservation of this recognition pattern.

## Material and methods

### Chemicals

The chemicals used for functional testing were purchased from Bachem (l-Trp-Trp-Trp, Cat# 4005236), Calbiochem (cholic acid, Cat# 2290101), and Sigma-Aldrich (l-Trp, Cat# T0254; d-Trp, Cat# T9753; glycocholic acid, Cat# G7132; flufenamic acid, Cat# F9005). Stock solution were either prepared in C1-buffer (130 mM NaCl (Carl Roth, Cat# 3957.1), 10 mM HEPES (PAN BioTech, Cat# P05-01500) pH 7.4, 5 mM KCl (VWR, Cat# 26764.232), 2 mM CaCl_2_ (neoFroxx, Cat# LC-5912.3) 0.18% glucose (VWR, Cat#101174Y) or in DMSO (Thermo Fisher Scientific, Cat# 11365058). The final DMSO concentration was kept to 0.5% or below. The applied concentrations were chosen based on previous experiments [30–32].

### Plasmids

The expression constructs for the human [18, 33], mouse [34], chicken [9], frog [9], and fish [13] bitter taste receptors were available from previous studies. The cDNAs of the receptors were extended by sequences encoding amino terminal sst3-export tags and carboxy terminal herpes simplex virus glycoprotein D (hsv)-tags. The point-mutated constructs coding for TAS2R14 mutants were generated previously [35].

### Cultivation of cells

HEK 293t-Gα16gust44 cells were grown in a monolayer in Dulbecco´s Modified Eagle Medium (DMEM) (Thermo Fisher Scientific, Cat# 41965-02), supplemented with 10% heat-inactivated fetal bovine serum (FBS) (Thermo Fisher Scientific, Cat# R-7988438.2), 1% l-glutamine (Sigma Aldrich, Cat# G7513) and 1% penicillin–streptomycin solutions (Sigma Aldrich, Cat# 15140-122), in culture dishes coated with 1 μg/mL poly-d-lysine (Thermo Fisher Scientific, Cat# 17318583). The cells were cultivated under 5% CO_2_ atmosphere at 37 °C and saturated air-humidity.

### Transfection

About 24 h before transfection, the HEK 293t-Gα16gust44 cells were seeded to reach ~ 60% confluence the next day in 10 μg/mL poly-d-lysine (Thermo Fisher Scientific, Cat# 17318583) coated clear bottom 96-well plates. The transfection was performed with 150 ng/well of plasmid DNA and 0.3 μL/well lipofectamine 2000 (Invitrogen, Cat# 11668–500) in serum-free DMEM as published before [36, 37]. As a negative control, an empty vector (mock) was transfected. After incubation for five hours at cell culture conditions, the medium was changed to supplemented DMEM.

### Calcium imaging

The HEK293T–Gα16gust44 cells were loaded with the fluorescence dye Fluo-4 AM (Abcam, Cat# ab241082), in combination with 2.5 mM probenecid (Sigma Aldrich, Cat# P8761) for 1 h in the dark at culture conditions as reported previously [9, 34]. Briefly, the cells were washed with C1 buffer and incubated for half an hour in the dark at room temperature. Directly before the measurement, the plate was washed again with C1 buffer. To apply agonists and to detect fluorescence changes a FLIPR^TETRA^ system (Molecular Devices, San Jose, USA) was used. As a cell viability control, somatostatin 14 (Bachem, Cat# 4033009) at a final concentration of 100 nM was employed. For calculations of ΔF/F values, the fluorescence changes observed for receptor transfected cells were mock subtracted using the corresponding identically treated cells transfected with empty vector. The data from three independent experiments, each performed in duplicates were checked for statistical significance using student’s *t*-test (two-sided; *p* < 0.05).

### Bioinformatics

The 25 human TAS2Rs [18], 35 mouse Tas2rs [34], 3 chicken Tas2rs [9], 2 turkey Tas2rs [9], 6 frog Tas2rs [9], and 7 zebrafish Tas2r [13] amino acid sequences were selected from previous publications and aligned using the software CLC MainWorkbench 23.0.4 with a gap creation penalty of 10.0 and a gap extension penalty of 1.0. For tree generation the neighbour joining algorithm, which can be used for tree construction [38], with Kimura correction and 1000 bootstrap replications were chosen.

### Molecular modelling

The tool ‘Develop Pharmacophore Model’ (Schrödinger Release 2022-3: Phase, Schrödinger, LLC, New York, NY, 2022) was used to generate pharmacophore models that match at least 75%. The model with the best PhaseHypo score was selected for the analysis. The 3D structure of TAS2R14 was modelled with Prime (Schrödinger Release 2022-3: Prime, Schrödinger, LLC, New York, NY, 2022) using the coordinates of TAS2R46 (PDB ID: 7XP6) as template (target-template sequence identity: 43%). The TAS2R14 receptor model is available at https://github.com/dipizio/TAS2R-models. Coordinates of glycocholic acid and l-Trp-Trp-Trp were retrieved from previous works [31, 39]. The ligand structures were prepared with LigPrep (Schrödinger Release 2022-3: LigPrep, Schrödinger, LLC, New York, NY, 2022.) at pH 7 ± 1. Induced Fit Docking (Schrödinger Release 2022-3: Glide and Prime, Schrödinger, LLC, New York, NY, 2022) simulations were performed to investigate the binding modes of the ligands. The box center was set within the centroid of ligand and the residues W66, L85, N87, W89, T90, N93, S183, Y240, A241 were selected as flexible residues.

## Results

### The bitter peptide l-Trp-Trp-Trp and bile acids concentration-dependently activated the same set of TAS2Rs

**Fig. 1:**
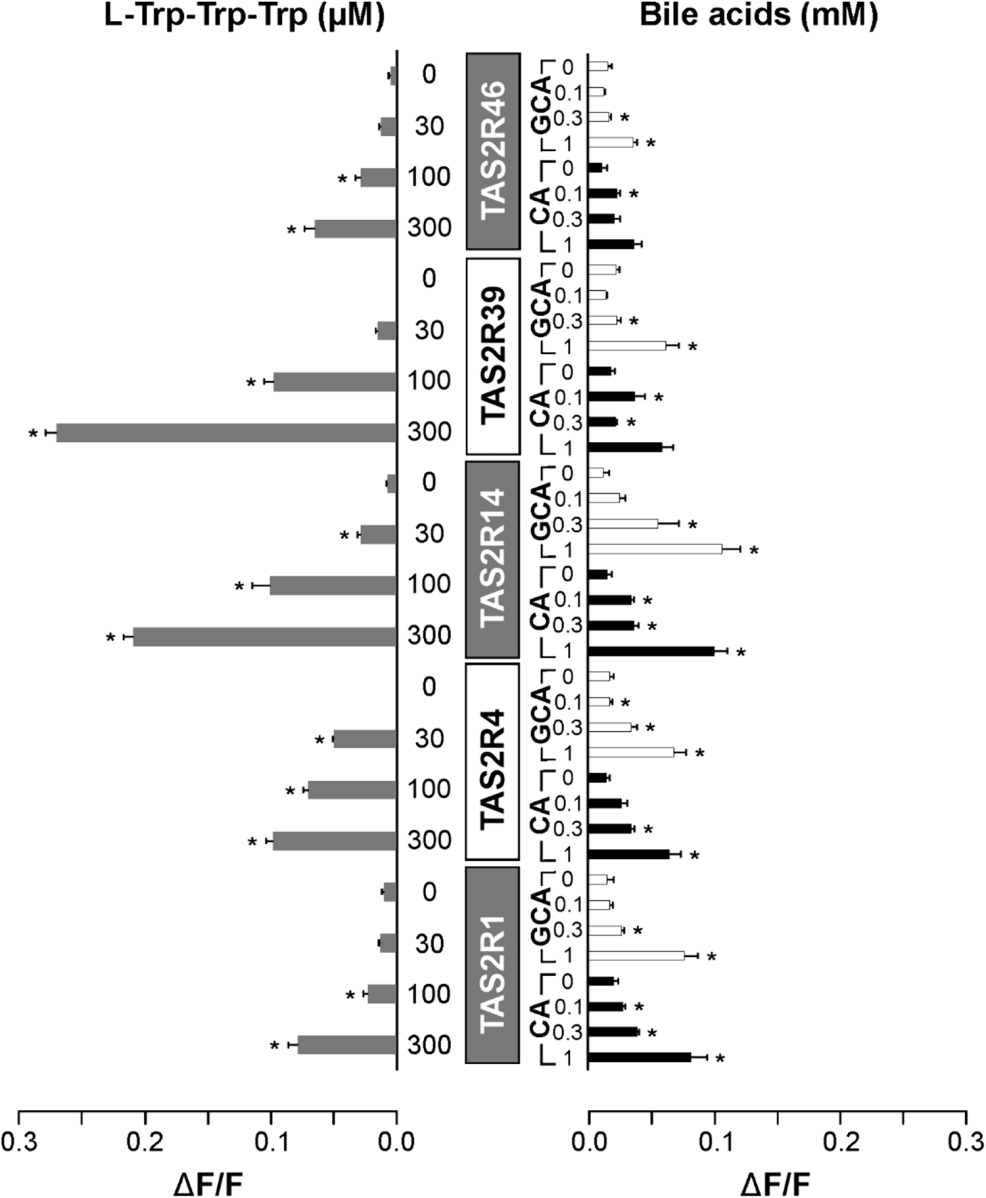
Human TAS2Rs response to bitter peptides and bile acids. The human bitter taste receptors TAS2R1, TAS2R4, TAS2R14, TAS2R39, and TAS2R46 were transiently transfected into HEK 293T-Gα16gust44 cells and subjected to functional calcium-mobilization experiments using a FLIPR^TETRA^. The receptors were stimulated either with l-Trp-Trp-Trp (gray bars, left side of panel) or the bile acids cholic acid (CA, black bars) and glycocholic acid (GCA, white bars) (right side of panel). The concentrations used for stimulation are depicted on the left (l-Trp-Trp-Trp in µM) and right (CA and GCA in mM) columns. The corresponding TAS2Rs are indicated in the center of the figure. Cells transfected with empty vector served as negative controls (see supplementary information). The relative changes in fluorescence upon substance application (ΔF/F) were monitored (scale bars are shown at the bottom left and right). Data represent mean ± SEM of three independent experiments, each performed in duplicates. *p < 0.05, (two-sided) student’s *t*-test

As a first approach to study the overlap in bitter taste receptor activation of bile acids and amino acids, we performed functional experiments using the five TAS2Rs activated by both natural compound groups, bile acids and peptides. The receptors TAS2R1, TAS2R4, TAS2R14, TAS2R39, and TAS2R46 were transiently expressed in HEK 293 t-Gα16gust44 cells and subjected to functional calcium-mobilization experiments. As test stimuli we used the peptide l-Trp-Trp-Trp, which was found to most robustly activate all 5 TAS2Rs [30], as well as the bile acids cholic acid and glycocholic acid [31]. An overview of the receptor responses is depicted in Fig. 1 (cf. Fig. S1A for data including stimulation of mock transfected cells).

Our results unambiguously show that TAS2R1, -R4, -R14, -R39, and -R46 all responded to l-Trp-Trp-Trp (Fig. 1), which corroborates previous findings [30]. While the signal magnitudes observed for TAS2R39 and TAS2R14 stand out, the pronounced response of TAS2R4 transfected cells stimulated with 30 µM l-Trp-Trp-Trp indicates the high sensitivity of this receptor for bitter peptides. The responses of the five receptors stimulated with either CA or GCA appear comparable in magnitude and apparent concentration-dependence suggesting roughly similar efficacies and potencies of conjugated and unconjugated cholic acid. Hence, it can be concluded that both types of stimuli, peptides and bile acids, represent agonists of comparative strengths.

**Fig. 2:**
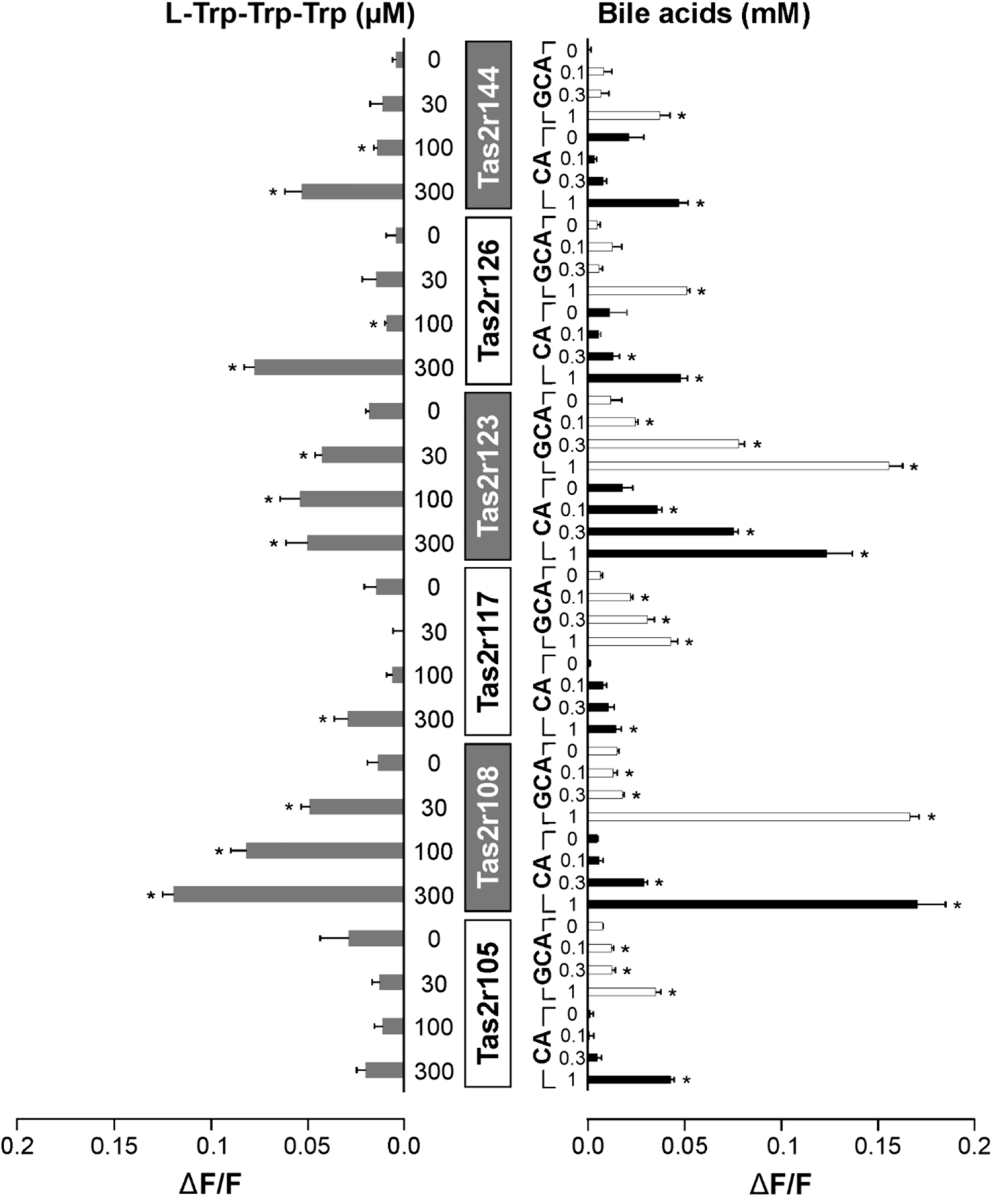
Mouse Tas2rs response to bitter peptides and bile acids. The mouse bitter taste receptors Tas2r105, Tas2r108, Tas2r117, Tas2r123, Tas2r126 and Tas2r144 were transiently transfected into HEK 293 T-Gα16gust44 cells and subjected to functional calcium-mobilization experiments using a FLIPR^TETRA^. The receptors were stimulated either with l-Trp-Trp-Trp (gray bars, left side of panel) or the bile acids cholic acid (CA, black bars) and glycocholic acid (GCA, white bars) (right side of panel). The concentrations used for stimulation are depicted on the left (l-Trp-Trp-Trp in µM) and right (CA and GCA in mM) columns. The corresponding Tas2rs are indicated in the center of the figure. Cells transfected with empty vector served as negative controls (see supplementary information). The relative changes in fluorescence upon substance application (ΔF/F) were monitored (scale bars are shown at the bottom left and right). Data represent mean ± SEM of three independent experiments, each performed in duplicates. *p < 0.05, (two-sided) student’s *t*-test

Recently, we demonstrated that a number of mouse receptors are activated by a variety of bile acids including cholic acid and glycocholic acid [31]. A comprehensive investigation with bitter peptides is lacking until now. To find out if also the mouse receptors respond to both types of stimuli, we functionally expressed the six bile acid responsive mouse bitter taste receptors Tas2r105, -r108, -r117, -r123, -r126, and –r144 and stimulated them with l-Trp-Trp-Trp as well as CA and GCA (Fig. 2; cf. Fig. S1 A for data including stimulation of mock transfected cells).

The results obtained for the mouse Tas2rs resemble those seen for the human receptors, although a somewhat more differentiated picture emerged. Whereas mouse receptors Tas2r108, Tas2r117, Tas2r123, Tas2r126, and Tas2r144 exhibit responses to l-Trp-Trp-Trp and the two bile acids, Tas2r105 only responded to the bile acids and not to the bitter tripeptide. Another important difference compared to the human receptors is the preference for bile acids over l-Trp-Trp-Trp of Tas2r123, whereas the Tas2r108 shows the opposite preference at least in terms of sensitivity. Nevertheless, the overall outcome of the mouse receptor experiment suggests a partial evolutionary conservation of bitter peptide and bile acid recognition in the mammalian order of euarchontoglires.

In order to elucidate if even more distantly related species share bile acid-bitter peptide activation in their bitter taste receptor repertoires, we screened receptors of chicken and frog with the same set of agonists.

**Fig. 3:**
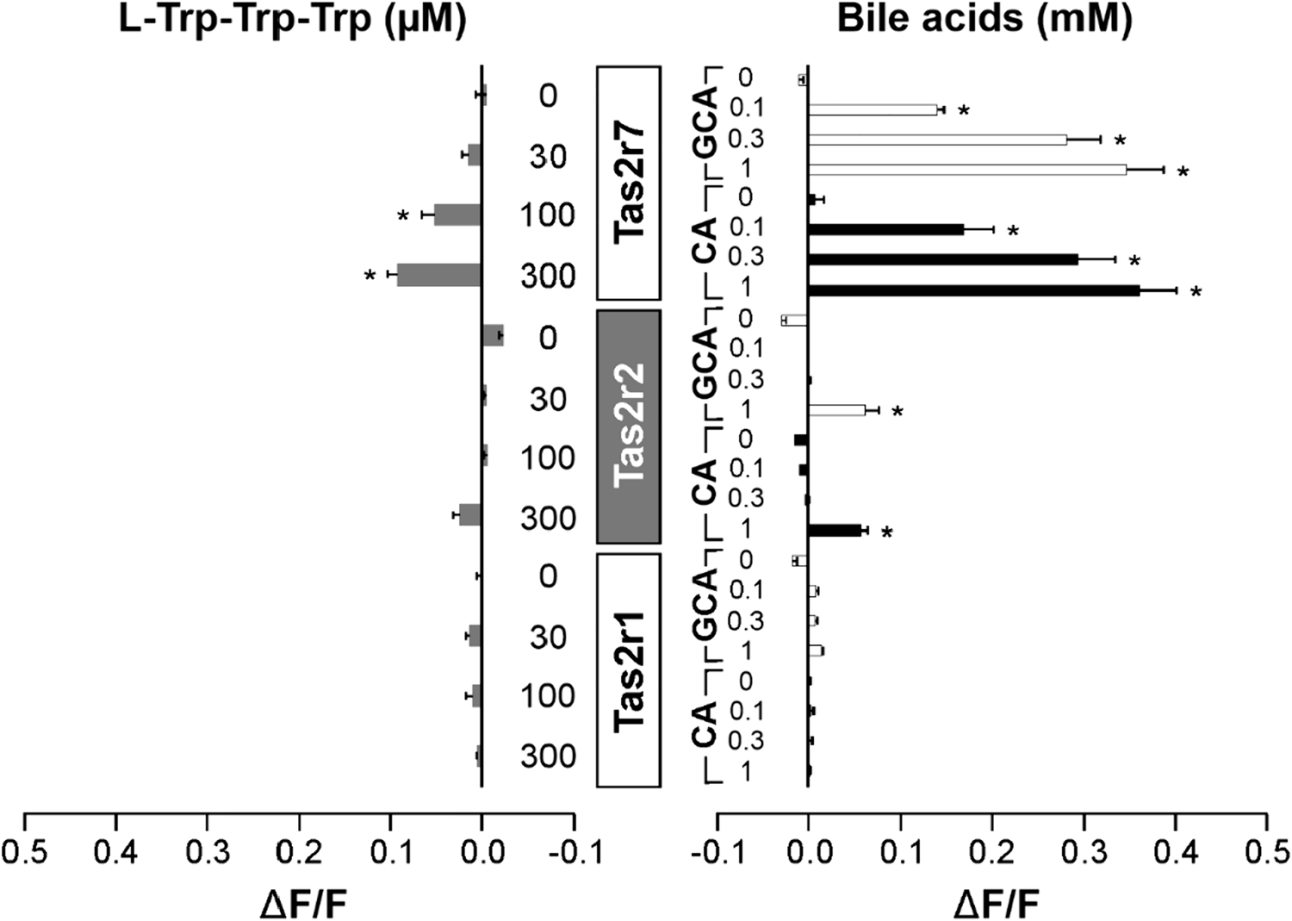
Chicken Tas2rs response to bitter peptides and bile acids. The chicken bitter taste receptors Tas2r1, Tas2r2, and Tas2r7 were transiently transfected into HEK 293T-Gα16gust44 cells and subjected to functional calcium-mobilization experiments using a FLIPR^TETRA^. The receptors were stimulated either with l-Trp-Trp-Trp (gray bars, left side of panel) or the bile acids cholic acid (CA, black bars) and glycocholic acid (GCA, white bars) (right side of panel). The concentrations used for stimulation are depicted on the left (l-Trp-Trp-Trp in µM) and right (CA and GCA in mM) columns. The corresponding Tas2rs are indicated in the center of the figure. Cells transfected with empty vector served as negative controls (see supplementary information). The relative changes in fluorescence upon substance application (ΔF/F) were monitored (scale bars are shown at the bottom left and right). Data represent mean ± SEM of three independent experiments, each performed in duplicates. *p < 0.05, (two-sided) student’s *t*-test

**Fig. 4:**
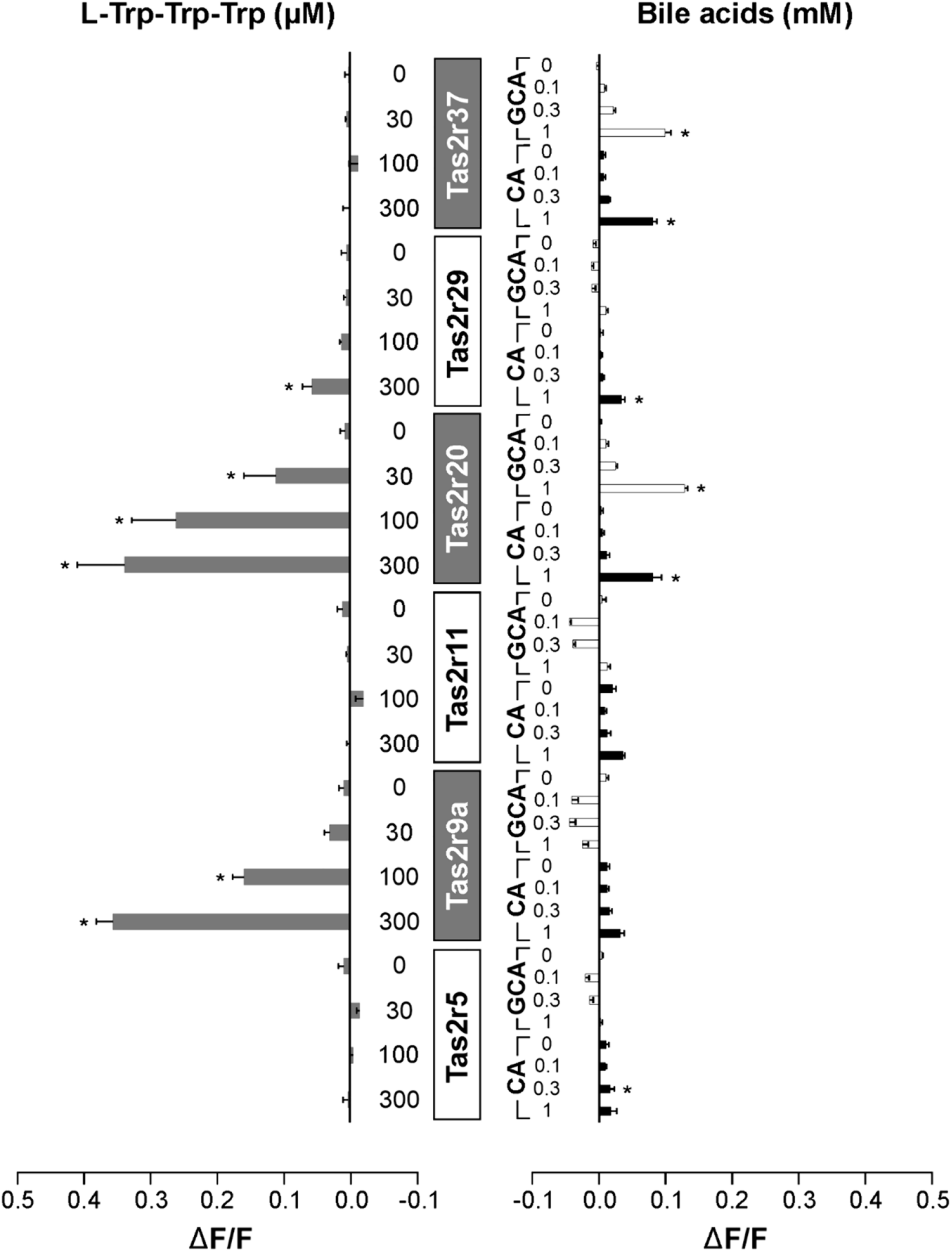
Frog Tas2rs response to bitter peptides and bile acids. The frog bitter taste receptors Tas2r5, Tas2r9a, Tas2r11, Tas2r20, Tas2r29, and Tas2r37 were transiently transfected into HEK 293T-Gα16gust44 cells and subjected to functional calcium-mobilization experiments using a FLIPR^TETRA^. The receptors were stimulated either with l-Trp-Trp-Trp (gray bars, left side of panel) or the bile acids cholic acid (CA, black bars) and glycocholic acid (GCA, white bars) (right side of panel). The concentrations used for stimulation are depicted on the left (l-Trp-Trp-Trp in µM) and right (CA and GCA in mM) columns. The corresponding Tas2rs are indicated in the center of the figure. Cells transfected with empty vector served as negative controls (see supplementary information). The relative changes in fluorescence upon substance application (ΔF/F) were monitored (scale bars are shown at the bottom left and right). Data represent mean ± SEM of three independent experiments, each performed in duplicates. *p < 0.05, (two-sided) student’s *t*-test

We found that the most broadly tuned chicken receptor Tas2r7 exhibited responses to l-Trp-Trp-Trp as well as to CA and GCA, with more pronounced responses for the two bile acids (Fig. 3; cf. Fig. S1 A for data including stimulation of mock transfected cells). The responses of chicken Tas2r2 were only significant for the highest concentrations of the two bile acids, whereas the response to l-Trp-Trp-Trp failed to reach statistical significance. In contrast to this, chicken Tas2r1 showed no response to both types of stimuli. These findings provide evidence that shared bile acid-bitter peptide responses existed even longer during the evolution of bitter taste receptors. Since the so far documented conservation of bitter taste receptor co-activation points to the existence of receptors with shared recognition spectra in the reptilian branch, we next investigated whether already amphibian species might have acquired such features. Therefore, we used the six bitter taste receptors of the Western clawed frog (*Xenopus tropicalis*), Tas2r5, Tas2r9a, Tas2r11, Tas2r20, Tas2r29, and Tas2r37 for further functional experiments (Fig. 4; cf. Fig. S1 A for data including stimulation of mock transfected cells).

Intriguingly, also one of the 6 frog receptors showed prominent responses to both types of stimuli, the Tas2r20, whereas the Tas2r29 exhibited only weak, yet significant, responses to l-Trp-Trp-Trp as well as to CA and GCA. The Tas2r9a was pronounced, but solely activated by l-Trp-Trp-Trp, whereas Tas2r37 responded exclusively to the two bile acids. For the remaining two receptors, Tas2r5 and Tas2r11, we could not observe activation by either stimuli.

As we also observed previously responses of bony fish bitter taste receptors to bile acids [13], we tested the zebrafish receptor Tas2r4 for an activation by l-Trp-Trp-Trp but did not obtain responses. Subsequent testing with l- and d-Trp revealed responsiveness to these amino acids suggesting rudimentary responsiveness to both substance classes (Fig. S1B).

### Structural similarity between l-Trp-Trp-Trp and glycocholic acid

**Fig. 5:**
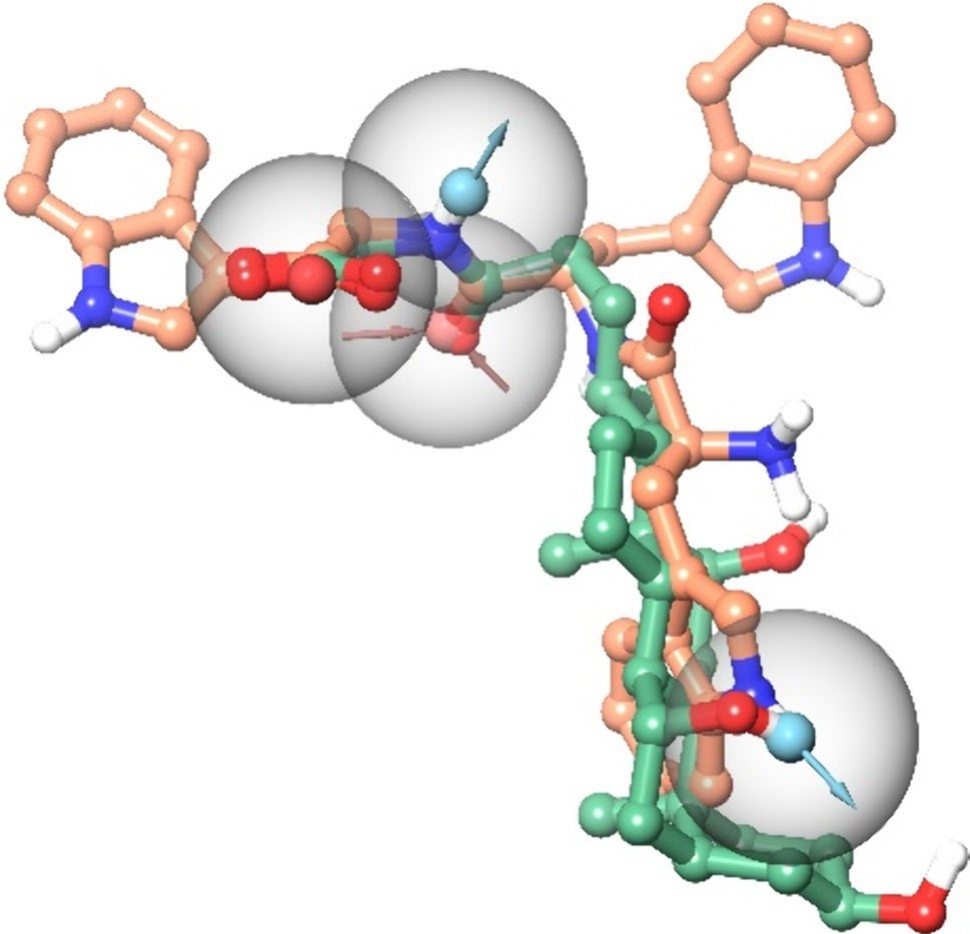
Pharmacophore Model (ADDN, PhaseHypoScore: 0.903) generated using the structures of l-Trp-Trp-Trp (in CPK ball&stick, with carbons in light orange) and glycocholic acid (in CPK ball&stick, with carbons in green). Pharmacophore features are reported as transparent spheres, blue and red spheres&vectors indicate H-bond donors and acceptors, respectively, and red spheres indicate negative charges

One possible and most simple reason for the frequent coincidence of l-Trp-Trp-Trp and bile acid activation of bitter taste receptors would be the existence of 3-dimensional structural similarities resulting in highly similar pharmacophores. To test this hypothesis, we generated pharmacophore models shared among l-Trp-Trp-Trp and GCA. In the best model (Fig. 5), the structure of the glycocholic acid aligns to the core of l-Trp-Trp-Trp to overlay three functional groups. The hydroxyl group at position 7 of glycocholic acid and the nitrogen of the indole of the first tryptophan of l-Trp-Trp-Trp can function as hydrogen bond donors, while the carboxy tail of the glycocholic acid aligns well with the peptide backbone of the C-terminus of l-Trp-Trp-Trp, so that the two molecules share three functional groups (H-bond acceptor, H-bond donor and one negatively charged group) in this region. The alignment provided by the pharmacophore suggests that two indole groups of the l-Trp-Trp-Trp should accommodate subpockets of the binding site that are not occupied by the glycocholic acid.

### Bitter peptide and bile acid responsiveness is phylogenetically conserved

**Fig. 6:**
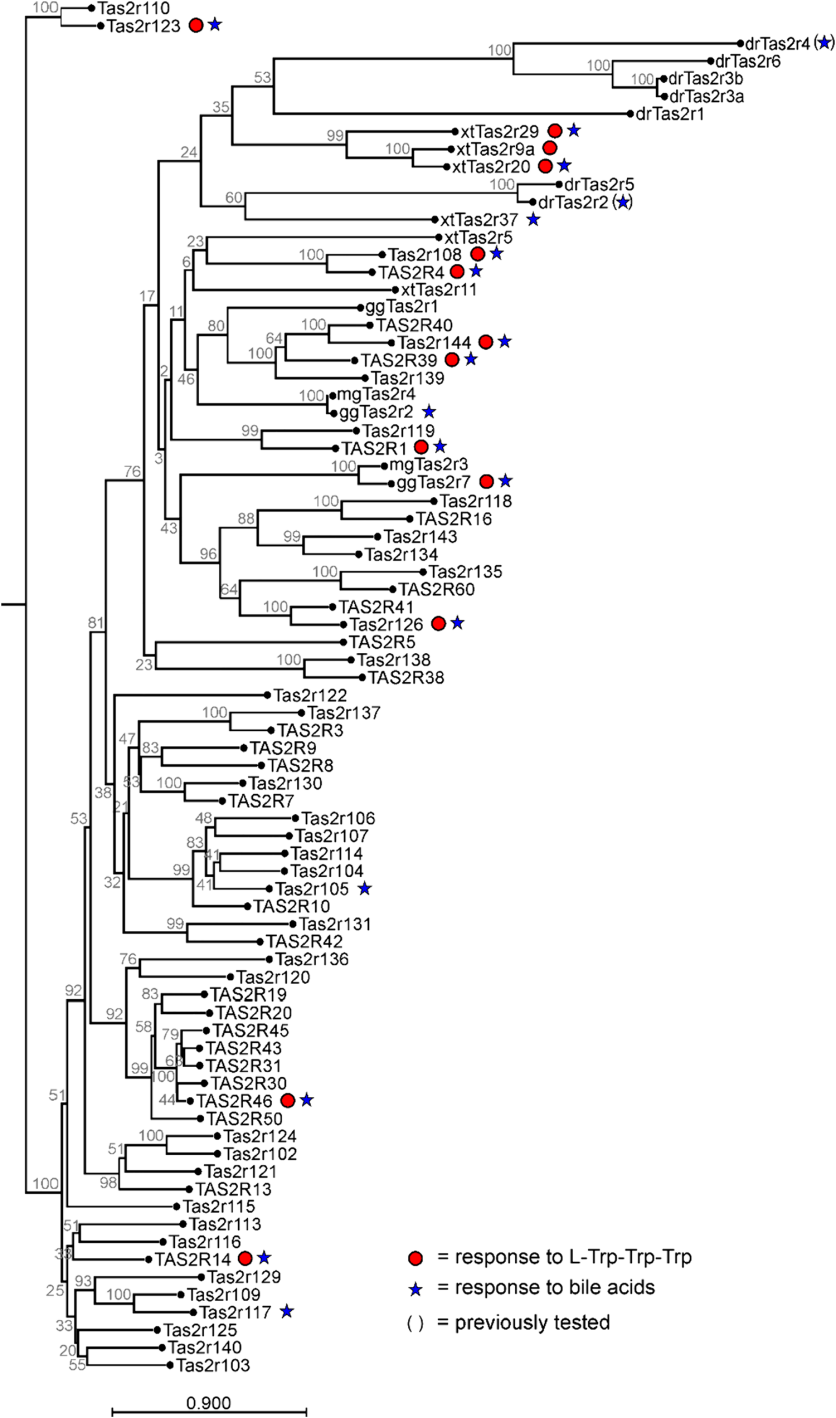
Phylogenetic tree of vertebrate bitter taste receptors and their responsiveness to l-Trp-Trp-Trp and bile acids. The 25 human (TAS2R_(number)_), 35 mouse (Tas2r_(number)_), 3 chicken (ggTas2r_(number)_), 2 turkey (mgTas2r_(number)_), 6 frog (xtTas2r_(number)_), and 7 zebrafish (drTas2r_(number)_) bitter taste receptor amino acid sequences were taken from previous publications. The bootstrap values (1000 replications, gray numbers) for the nodes are given in %. Statistically significant responses to l-Trp-Trp-Trp (red dot), bile acids (star) are labelled. Symbols in parenthesis indicate data taken from [13]. Scale bar = amino acid substitutions per site

Another reason for the shared receptor patterns of these compounds could be the evolutionary conservation of bitter taste receptor activation by the two compound groups. We therefore performed a phylogenetic analysis of vertebrate bitter taste receptors. The result is shown in Fig. 6.

As evident from the phylogenetic tree of human, mouse, bird (chicken, turkey), frog, and zebrafish receptors, responsiveness to bitter peptides and/or bile acids is rather distributed over the array of aligned receptors. Nevertheless, quite a number of receptors with the investigated activities cluster together suggesting the possibility for evolutionary conservation. As this cluster contains receptors of human, mouse, birds, frog, and fish and hence, covers a long evolutionary period this could hint at a rather early development of bitter peptides and bile acid sensitivity, or as hypothesized before, the existence of endogenous receptor-ligand functions.

### Similar binding modes of l-Trp-Trp-Trp and glycocholic acid within the TAS2R14 binding site

**Fig. 7:**
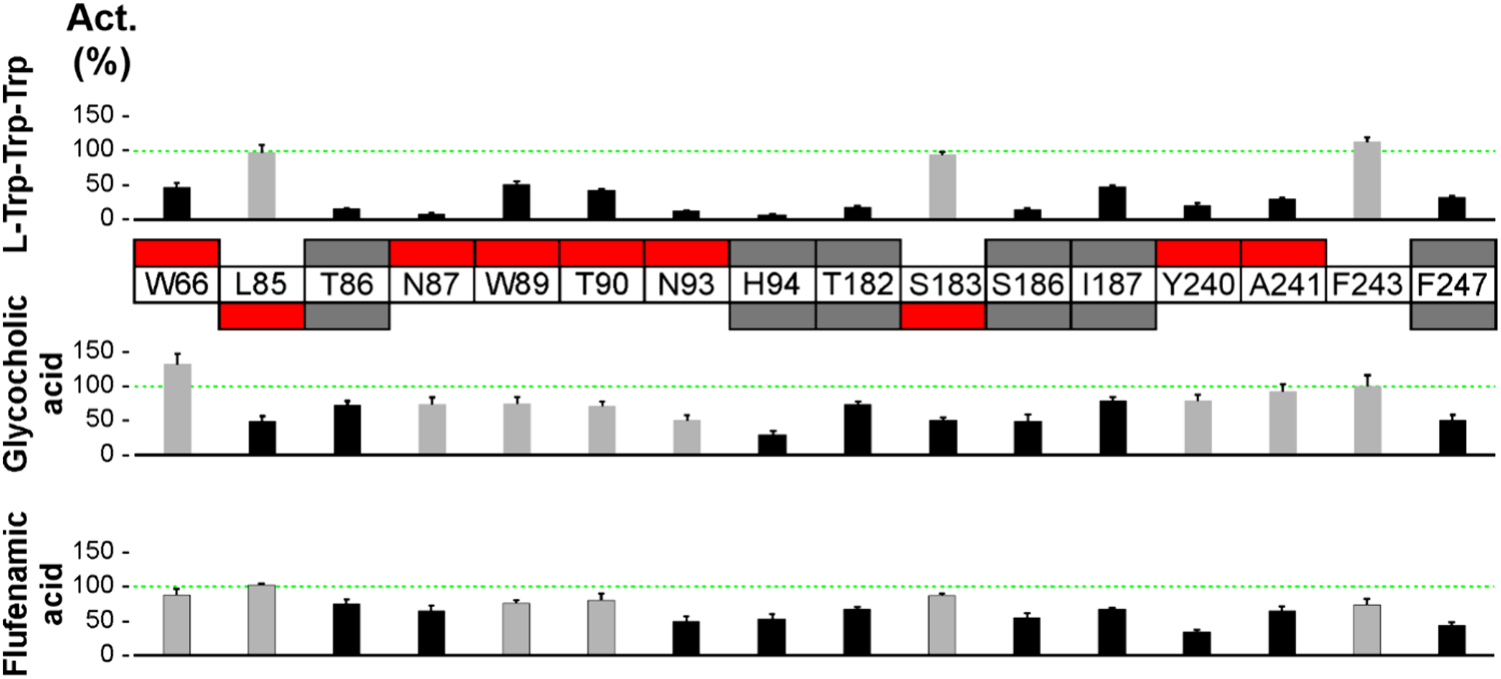
Mutated TAS2R14 responds partially differential to l-Trp-Trp-Trp and glycocholic acid. The mutant positions are indicated between the *l-*Trp-Trp-Trp and glycocholic acid graphs. Green dashed lines refer to responses obtained for wildtype TAS2R14 set to 100%. Red labeled boxes point to statistically significant changes in receptor activation if at least one compound including flufenamic acid was not affected by the mutation and thus the receptor is fully functional. Gray labeled boxes point to statistically significant changes in receptor activation in cases where all compounds were affected and hence, global changes in receptor activation cannot be excluded. For *l-*Trp-Trp-Trp (upper graph) and glycocholic acid (middle graph) black bars indicate statistically significant changes and gray bars no significance. Flufenamic acid (lower graph; black bars = statistically significant changes, gray bars = no significance) was used as an unrelated TAS2R14 agonist. Data represent mean ± SEM of three independent experiments, each performed in duplicates. *p < 0.05, (two-sided) student’s *t*-test

Ligand recognition is a complex process. A ligand can bind to different proteins using different interaction patterns, and chemically diverse compounds can use similar interaction hotspots to interact with the same protein [40]. For example, diverse proteins in complex with bile acids are reported in the Protein Data Bank, but they show different profiles of ligand–protein interactions [41–43]. In order to find out if the binding modes of l-Trp-Trp-Trp and bile acids to responsive TAS2Rs show similarities, we employed functional experiments with mutated TAS2R14 constructs available from a comprehensive previous structure–function study (Fig. 7).

The functional experiments were performed with 16 point-mutated constructs of human TAS2R14 using l-Trp-Trp-Trp and glycocholic acid as bile acid stimulus. Cholesterol, the precursor for bile acid synthesis was identified as TAS2R14 agonist in this study (see supplementary Fig. S2), however, due to the very weak agonistic activity not included. As an unrelated reference stimulus we included flufenamic acid in this set of experiments. The outcome of the experiment is depicted in Fig. 7. We observed that the number of mutations affecting l-Trp-Trp-Trp exceeds the number of those affecting glycocholic acid. Whereas mutated positions W66A, N87A, W89A, T90A, N93A, Y240A, and A241I led to reduced l-Trp-Trp-Trp responses, only the mutations L85A and S183A led to reductions in glycocholic acid responses. A single mutated construct, F243A, exhibited unchanged activations by all agonists including flufenamic acid, whereas the constructs TAS2R14-T86A, -H94A, -T182A, -S186A, -I187A, and -F247A showed reduced activations by all agonists. While it cannot be excluded that the latter mentioned set of mutants suffer from general impairment in responsiveness, it needs to be stated that flufenamic acid also depends on specific interactions with the receptor and hence, it is possible that all three agonists are specifically affected by some of the mutant positions. In conclusion this experiment revealed that l-Trp-Trp-Trp and glycocholic acid do not share an identical set of contact points to activate TAS2R14.

### Predicted binding modes of l-Trp-Trp-Trp and glycocholic acid within the TAS2R14

**Fig. 8:**
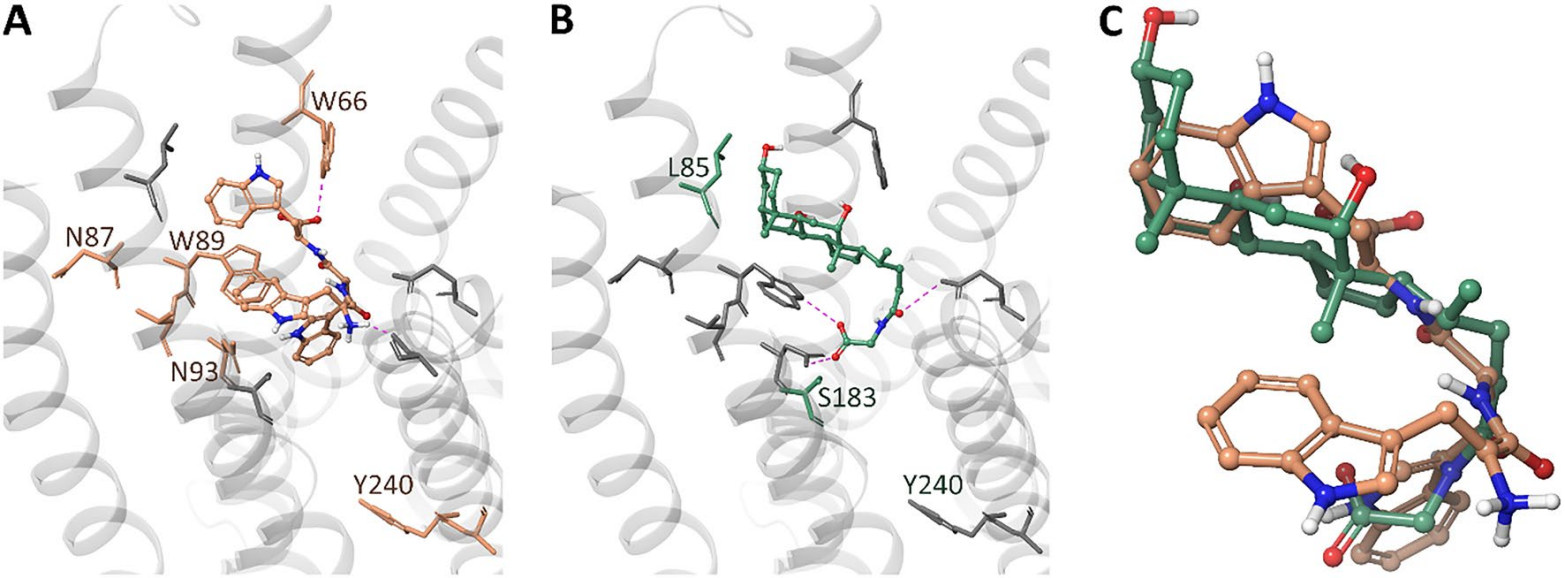
Putative binding modes of l-Trp-Trp-Trp (**A**) and glycocholic acid (**B**) into the TAS2R14 binding site. A zoom-on on the structural alignment of the docking poses is reported in panel C. Glycocholic acid and l-Trp-Trp-Trp structures are shown as CPK ball&stick with carbons in light orange and green, respectively. Binding site residues analysed by mutagenesis are shown as sticks. *l-*Trp-Trp-Trp specific residues are colored in light orange and GCA specific residues in green. Hydrogen bonds are displayed as magenta dashed lines

Using modeling and docking calculations, we investigated the possible binding modes of l-Trp-Trp-Trp and GCA within the binding site of TAS2R14. We used a structural model of TAS2R14 built on the solved structure of TAS2R46 (PDB ID: 7XP6, sequence identity 43%) and run induced-fit docking simulations to allow the flexibility of residues W66, L85, N87, W89, T90, N93, S183, Y240, and A241. The best poses obtained for l-Trp-Trp-Trp and glycocholic acid are reported in Fig. 8. Both ligands bind to the orthosteric binding site between TM2, TM3, TM5, TM6 and TM7. l-Trp-Trp-Trp inserts the first tryptophan residue between W66 and W89, the second tryptophan in the bottom of the pocket, pointing to Y240, and the third tryptophan between TM3 (close to W89 and N93) and TM5. The glycocholic acid binds pointing its carboxy group deep in the binding site and interacting with N83, while its hydrophobic backbone sits in TM3, accommodated in a subpocket formed by L85 and F82. Within the binding site, the compounds do not align as suggested by the pharmacophore model. Glycocholic acid shares the portion of the binding pocket occupied by the second and third tryptophan residues (Fig. 8C). The region that is occupied by l-Trp-Trp-Trp but not by GCA includes residues N87, W89, T90 and N93, which were shown as l-Trp-Trp-Trp specific residues in the mutagenesis experiments. Also, l-Trp-Trp-Trp is predicted to enter deeper in the binding site, and this could explain the different effect of the Y240A and A241I mutants towards the two ligands.

## Discussion

Following up on previous publications showing the activation of human TAS2Rs by bitter amino acids and peptides [30] and the activation of human and mouse bitter taste receptors by bile acids [31], we here investigated the overlap of both compound classes on the same subset of receptors. We demonstrated the complete overlap of the bitter peptide l-Trp-Trp-Trp and the bile acids cholic acid and glycocholic acid on five human and six mouse bitter taste receptors. The finding that cross-detection of peptides and bile acids by bitter taste receptors is widespread and extends from the tested mammalian species over birds to frogs raises the question if this phenomenon developed several times independently during evolution or if this feature might be conserved. Our phylogenetic analyses (Fig. 6) shows that likely both explanations need to be taken into account. Some of the responding receptors occur well separated from each other within the phylogenetic tree and therefore, the co-sensitivity may have evolved independently. As bile acids are rigid structures, the flexible peptide chain present in l-Trp-Trp-Trp could adopt a similar structure with agonistic properties and hence, this “structural mimicry” is causing co-activation of those receptors.

The clustering of numerous bitter taste receptors of human, mouse, chicken, and frog in the upper part of the phylogenetic tree (Fig. 6) suggests that for these receptors the co-sensitivity might originate from a common ancestral receptor. In light of the strong functional conservation of phylogenetically old bitter taste receptors such as in sharks [44] and bony fish [13], a selective pressure originating from the need to maintain an extra-gustatory function, e.g. to detect endogenous agonists such as bile acids, could counteract the rapid evolutionary development of xenobiotics-directed detection properties and hence, a gene sharing mechanism similar to crystallin genes [45] could exist. Although the authors are not aware of similarly striking overlaps of bitter taste receptor agonist profiles, it appears likely that additional cases may emerge in the future. Alternatively, the fact that many bitter agonists activate multiple bitter taste receptors and many bitter taste receptors detect the same agonist [18], may obscure already existing similar cases because of lower receptor numbers affected. The increasing use and precision of machine learning applications for the prediction of bitter ligands [46–48] may allow detection of also less obvious cases.

While the conservation of l-Trp-Trp-Trp and bile acid bitter detection by the taste systems of distantly related species is intriguing, it is noteworthy that the physiological impacts of these compounds exhibit pronounced species differences (for a comprehensive review see [49]). With respect to l-Trp, some species are rather sensitive and suffer toxic effects from high blood tryptophan levels, whereas other species tolerate much higher levels. Tryptophan tolerance is strongly affected by blood albumin concentrations, which binds tryptophan and lowers free circulating tryptophan levels [49], as well as by the activity of tryptophan degrading enzymes such as hepatic Trp 2,3-dioxygenase [50, 51]. Among the species investigated in this study human, mouse, chicken, turkey can be considered tryptophan tolerant, whereas some frog and most fish species can be quite sensitive to elevated blood tryptophan levels mostly due to the absence of albumin in the blood [49]. As most of the species investigated in this study tolerate tryptophan well, it seems unlikely that the conserved taste sensitivity to l-Trp-Trp-Trp is linked to toxicity, however, future research directly targeting the relationship of taste sensitivity and tryptophan toxicity could be revealing. In this regard, also species differences in bile acid metabolism and toxic effects and their correlation with taste sensitivities are of considerable interest. It has been shown that hepatotoxicity of bile acids fed to rodents correlates roughly with hydrophobicity [52] and is modulated by the metabolic ability for detoxification [53]. In our previous study on bitter receptor activation by a variety of bile acids, we have not observed a correlation of bile acid hydrophobicity and their potencies to induce bitter taste receptor responses [31], but again, more detailed future studies seem warranted.

Our findings shed new light on the somewhat surprising bitterness of some amino acids and peptides among which numerous essential amino acids are found. In light of the original assumed warning function of bitter taste, a rejection of essential amino acids or energy-rich peptides seems counter-intuitive. However, most l-amino acids in unprocessed food items occur as proteins, which rarely possess a taste except for some sweet plant proteins such as brazzein [54]. It is rather the decomposition of food accompanied by proteolytic digestion that liberates l-amino acids and peptides from their precursor proteins to generate bitter breakdown products, and hence a gustatory alert from naturally decaying food sources seems warranted, although controlled fermentations are historically widely used for food processing. With regard to the current study, the structural similarities of bile acids with some amino acids and peptides such as l-Trp-Trp-Trp may come with a trade-off, namely the cross-reactivity with bile acid’s bitterness. As bile acids are neither frequently consumed by animals nor would bile acids be considered particularly poisonous and thus require taste-based rejection behavior one may speculate that the nature of bile acids as endogenous ligands of bitter taste receptors may account partly for the development of sensitive bitter taste receptors.

## Acknowlegdements

The authors thank Catherine Delaporte for expert technical assistance.

### Supplementary Information

Below is the link to the electronic supplementary material.

Supplementary file1 (DOCX 471 KB)

Supplementary file2 (DOCX 70 KB)

## Data Availability

All data supporting the results of this article are provided in the article and on-line supplementary data.
