# Supplementary material for: Membrane-bound chemoreception of bitter bile acids and peptides is mediated by the same subset of bitter taste receptors
Source: Cell Mol Life Sci. 2024 May 15;81(1):217. doi: 10.1007/s00018-024-05202-6 (PMC11096235; doi:10.1007/s00018-024-05202-6)
Supplement: Supplementary file 1 — Supplementary file1 (DOCX 471 KB) [file 18_2024_5202_MOESM1_ESM.docx]

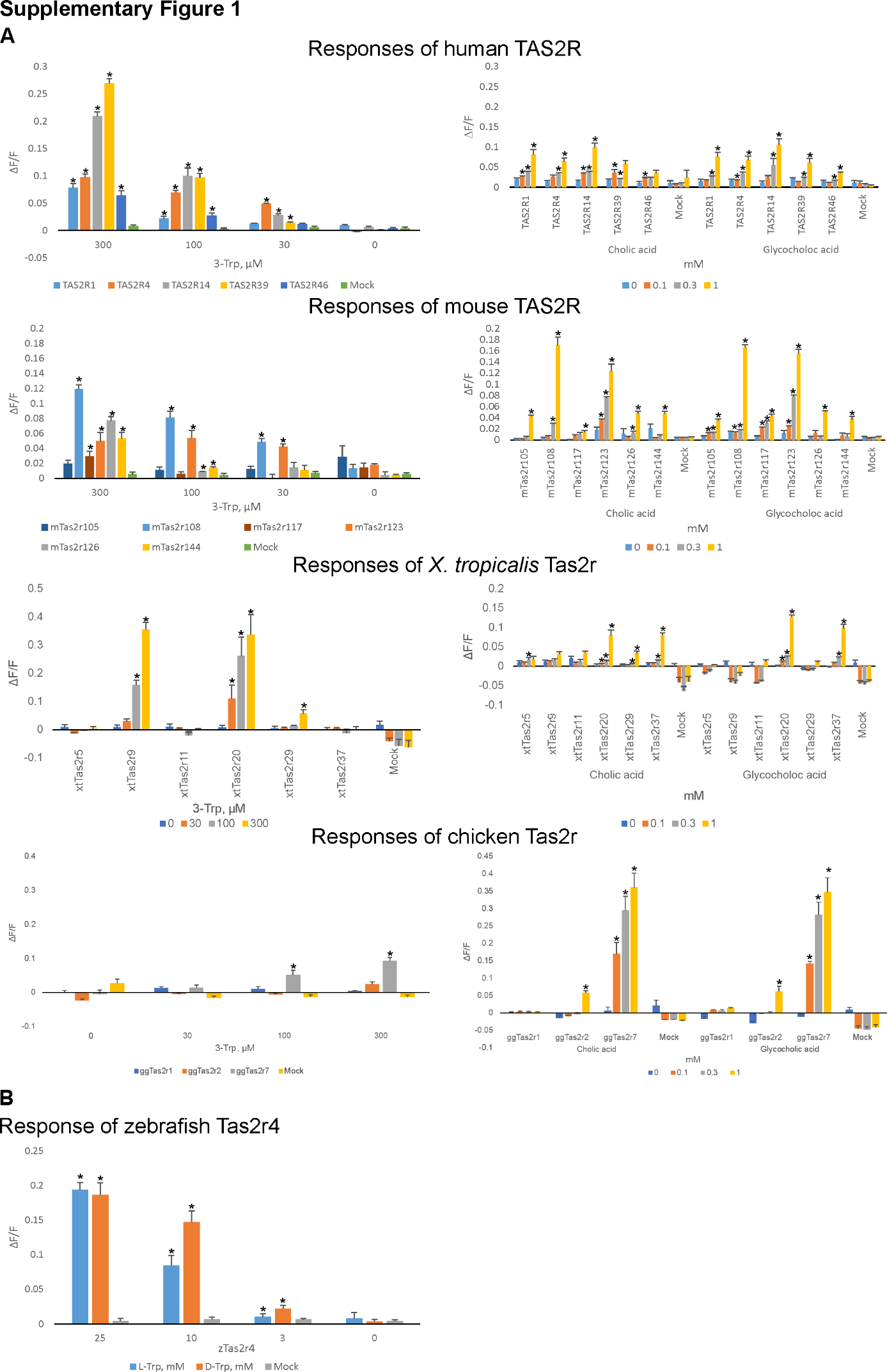


**Supplementary Figure 1.** *Extended response properties of bitter taste receptors of different vertebrate species.* **A)** The human, mouse, frog (X.tropicalis), and chicken bitter taste receptors were transiently transfected into HEK 293T-Gα16gust44 cells and subjected to functional calcium-mobilization experiments using a FLIPR^TETRA^. The receptors were stimulated either with L-Trp-Trp-Trp (gray bars, left side of panel) or the bile acids cholic acid (CA, black bars) and glycocholic acid (GCA, white bars) (right side of panel). The concentrations used for stimulation are depicted at the bottom of the corresponding panels (L-Trp-Trp-Trp in µM; CA and GCA in mM). The corresponding TAS2Rs are indicated in the center of the figure. Cells transfected with empty vector served as negative controls (= Mock). The relative changes in fluorescence upon substance application (ΔF/F) were monitored (scale bars are shown at the left). **B)** Response properties of zebrafish receptor zTas2r4. The bile acid-sensitive bitter taste receptor zTas2r4 did not respond to L-Trp-Trp-Trp, but as shown here, a response to L- and D-Trp was evident. Data represent mean ± SEM of three independent experiments, each performed in duplicates. *, p<0.05, (two-sided) student's t-test.
