# Supplementary material for: Membrane-bound chemoreception of bitter bile acids and peptides is mediated by the same subset of bitter taste receptors
Source: Cell Mol Life Sci. 2024 May 15;81(1):217. doi: 10.1007/s00018-024-05202-6 (PMC11096235; doi:10.1007/s00018-024-05202-6)
Supplement: Supplementary file 2 — Supplementary file2 (DOCX 70 KB) [file 18_2024_5202_MOESM2_ESM.docx]

**Supplementary Figure 2**

**
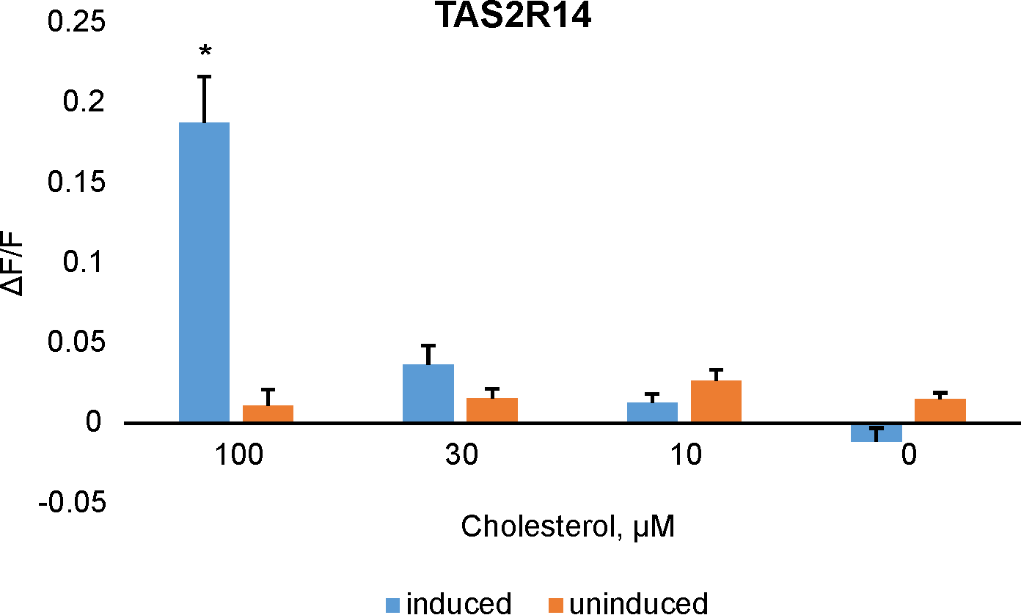
**

**Supplementary Figure 2.** *Human TAS2R14 responses to cholesterol.* HEK 293T-Gα16gust44 cells stably transfected with a tetracycline-inducible human TAS2R14 were subjected to functional calcium-mobilization experiments using a FLIPR^TETRA^. The induced TAS2R14 was stimulated with cholesterol at the indicated concentrations (blue bars). Uninduced cells (orange bars) served as negative controls (= Mock). The relative changes in fluorescence upon substance application (ΔF/F) were monitored (scale bars are shown at the left). Data represent mean ± SEM of three independent experiments, each performed in duplicates. *, p<0.05, (two-sided) student's t-test.
